# Crabohydrate-Related Inhibitors of Dengue Virus Entry

**DOI:** 10.3390/v5020605

**Published:** 2013-02-06

**Authors:** Kazuya I.P.J. Hidari, Tomoko Abe, Takashi Suzuki

**Affiliations:** Department of Biochemistry, School of Pharmaceutical Sciences, University of Shizuoka, and Global COE Program for Innovation in Human Health Sciences, 52-1 Yada, Suruga-ku, Shizuoka-shi, Shizuoka 422-8526, Japan; E-Mail: hidari@u-shizuoka-ken.ac.jp (K.I.P.J.H.)

**Keywords:** dengue virus, receptor, endocytosis, fusion, entry inhibitor, carbohydrate, glycosaminoglycan

## Abstract

Dengue virus (DENV), which is transmitted by *Aedes* mosquitoes, causes fever and hemorrhagic disorders in humans. The virus entry process mediated through host receptor molecule(s) is crucial for virus propagation and the pathological progression of dengue disease. Therefore, elucidation of the molecular mechanisms underlying virus entry is essential for an understanding of dengue pathology and for the development of effective new anti-dengue agents. DENV binds to its receptor molecules mediated through a viral envelope (E) protein, followed by incorporation of the virus-receptor complex inside cells. The fusion between incorporated virus particles and host endosome membrane under acidic conditions is mediated through the function of DENV E protein. Carbohydrate molecules, such as sulfated glycosaminoglycans (GAG) and glycosphingolipids, and carbohydrate-recognition proteins, termed lectins, inhibit virus entry. This review focuses on carbohydrate-derived entry inhibitors, and also introduces functionally related compounds with similar inhibitory mechanisms against DENV entry.

## 1. Introduction

Flaviviruses are enveloped viruses with an envelope (E) protein on the surface of the lipid bilayer membrane. Dengue virus (DENV), which belongs to the genus Flavivirus, family Flaviviridae, is transmitted from human to human by *Aedes* mosquitoes [1,2]. DENV causes febrile illness and more serious complications, such as hemorrhagic fever disease [3,4]. There are four virus serotypes, type 1 (DENV1) to type 4 (DENV4), which have similar clinical manifestations and epidemiology in tropical and subtropical regions of the world. At present, more than two billion people are at risk of infection [4,5,6,7]. A previous study demonstrated DENV tissue tropism in humans and mice where active DENV replication was occurring [8,9]. Virus antigen was detected in macrophages and dendritic cells of the spleen and lymph nodes of both host species. These cells migrate to the lymph nodes where DENV initially propagates and spreads to secondary replication tissues, such as bone marrow myeloid cells and hepatocytes in the liver [8]. To date, C-type lectins such as dendritic cell-specific ICAM3-grabbing non-integrin (DC-SIGN) and C-type lectin domain family 5 member A (CLEC5A) [10,11,12,13], mannose-receptor [14], glucose-regulating protein 78 (GRP78/Bip) [15], CD14 [16], heparan sulfate (HS) [17,18,19,20,21], and glycosphingolipids, such as neolactotetraosylceramide (nLc_4_Cer) [22,23], have been reported as putative receptor molecules for DENV. Most of these molecules are involved in carbohydrate-protein interaction. The structures of DENV E proteins have been elucidated by crystallography and NMR analyses [24,25,26,27]. These studies provided a structural basis for understanding the molecular mechanisms of virus entry.

**Figure 1:**
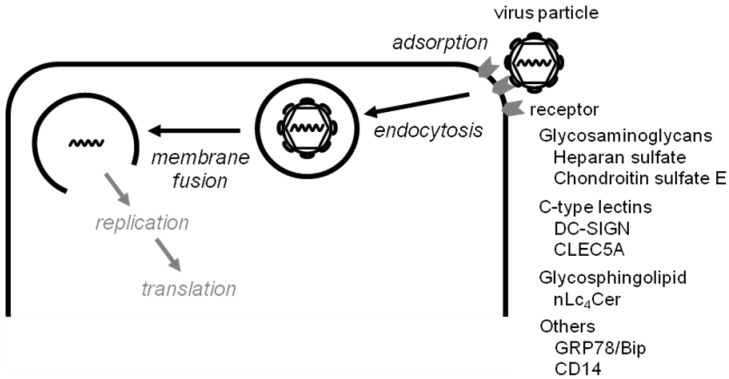
Endocytotic entry pathway of the enveloped virus life cycle.

DENV binds to as yet undefined receptor molecules on the host cell surface, followed by incorporation through the receptor-mediated endocytotic pathways. Fusogenic conformational changes in the virus envelope glycoprotein (E protein) are induced by the acidic environment of the endosome, resulting in fusion between virus particles and the host endosome membrane, and subsequent viral disassembly [28,29]. Single-stranded virus RNA with positive polarity, approximately 11 kb in length, which contains a single open reading frame encoding a polyprotein, is released into the cytoplasm and acts as a template for genome replication and protein translation events [27,30] (Figure 1). Many factors derived from host cells are thought to be involved in these entry processes of DENV infection. Several lines of evidence regarding host factors indicate that HS or the highly sulfated forms of glycosaminoglycans, such as chondroitin sulfate E (CSE), on the host cell surface are essential for the entry of flaviviruses including DENV [16,18,31,32,33,34,35]. An understanding of the molecular interactions mediated through virus envelope proteins in virus entry into the target cells is critical for elucidation of the mechanisms of virus tropism, such as host, tissue, and cell preferences.

Methods for the control and prevention of DENV by safe and long-lasting vaccination have not been established. Therefore, there is a requirement for effective antiviral agents and therapeutic concepts for DENV infection. However, at present, no specific treatments are clinically available for DENV infection. The virus entry process mediated through host carbohydrate molecule(s) is crucially involved in virus propagation and the pathological progression of dengue disease. Based upon the structures and functions of the carbohydrate molecules involved in DENV entry, several types of inhibitors that block DENV entry into cells have been generated [36]. This review article focuses on the chemical and biochemical properties of carbohydrate-derived inhibitors of DENV entry, such as GAGs, glycoproteins and glycosphingolipids, and also introduces functionally related inhibitors. 

## 2. Entry Inhibitors

Host factors and domains of virus E protein that are involved in DENV entry are likely to be useful targets in efforts to generate inhibitors of virus infection useful in both basic research and clinical medicine [37]. The early stages of infection by enveloped viruses, including DENV, involve two major processes—virus adsorption and fusion—for which target molecules may be useful for the development of antiviral agents (Figure 1). Here, we categorize and describe two types of entry inhibitor: 1) inhibitors of virus adsorption, and 2) inhibitors of virus-induced membrane fusion. 

### 2.1. Inhibitors of virus adsorption

**Figure 2:**
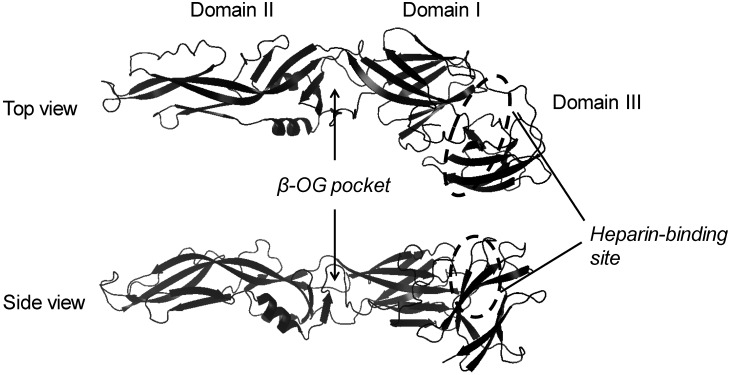
Structure of dengue virus (DENV) EGP. The structure of EGP based on the three-dimensional structure of PDB accession number 1OKE. The figure was prepared with PyMol.

Table 1 shows the chemical and antiviral properties of carbohydrate-mimetic inhibitors of DENV adsorption. Sulfated glycosaminoglycans, such as heparin, inhibit the early step of dengue virus infection through interaction with envelope (E) protein. Heparin binding sites for DENV consist of basic amino acid clusters on domain III, the putative receptor-binding domain in the crystal structure of the flavivirus E protein (Figure 2) [17,24,25,38,39]. Another GAG, chondroitin sulfate E (CSE), significantly reduced infectivity of all dengue virus serotypes toward BHK-21 and Vero cells. Virus binding to CSE or heparin was cross-inhibited by soluble CSE or heparin. It is suggested that common carbohydrate determinants on CSE and heparin could be essential epitopes for interaction of DENV, and may be responsible for DENV inhibition [35].

**Table 1:** Chemical and antiviral properties of inhibitors of virus adsorption

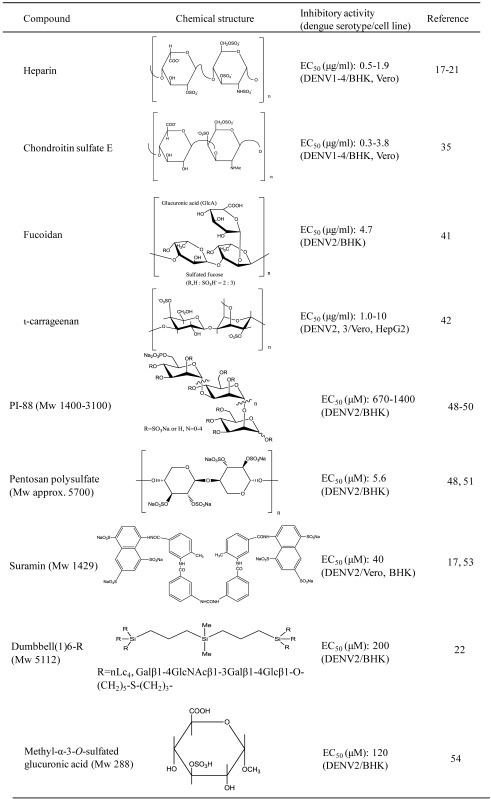

Sulfated polysaccharides obtained from the red seaweed *Gymnogongrus griffithsiae* and the marine alga *Cladosiphon okamuranus*, the kappa/iota/nu carrageenan derivative G3d, and fucoidan were shown to be selective inhibitors of DENV2/3 and 2 multiplications in cells, respectively [40,41,42]. G3d is an active DENV2/3 inhibitor that predominantly suppresses the initial processes of virus adsorption and internalization. Fucoidan is comprised of carbohydrate units containing glucuronic acid and sulfated fucose residues. This compound exclusively inhibits DENV2 infection. The infection was inhibited when the virus was pretreated with fucoidan. Structure-activity analysis demonstrated that glucuronic acid and the sulfated functional group from fucoidan were essential for inhibition of viral infection. The virus particles bound exclusively to fucoidan, indicating that fucoidan interacts directly with DENV2 E protein. Structure-based analysis suggested that Lys310 and Arg323 of DENV2 E protein, which are conformationally proximal to the putative heparin binding residues, are critical for the interaction with fucoidan. The variation in antiviral activity of two natural sulfated polysaccharides depends on the viral serotype. Many studies have demonstrated that diverse types of sulfated polysaccharides have anti-DENV activity [43,44,45,46,47].

The glycosphingolipid neolactotetraosylceramide (nLc_4_Cer) expressed on the cell surface of DENV-susceptible cells, human erythroleukemia K562, and baby hamster kidney BHK-21 is recognized by four serotypes of DENV. The non-reducing terminal disaccharide residue Galβ1-4GlcNAc of nLc_4_Cer is a critical determinant for the binding of DENV2. Chemically synthesized derivatives carrying multiple carbohydrate residues of nLc_4_, but not nLc_4_ oligosaccharide, inhibited DENV2 infection of BHK-21 cells [22].

The antiviral effects of heparan sulfate (HS) mimetics, such as suramin and pentosan polysulfate (PPS), and PI-88, have been reported against DENV. PI-88 is a mannose-containing di- to hexasaccharide with a high degree of sulfation [48,49,50]. PPS, a semi-synthetic β-d-xylopyranose polymer with a higher degree of sulfation than heparin, has been used for the prevention of postoperative thromboembolism and treatment of interstitial cystitis [48,51]. This compound also reduces virus infectivity by steric hindrance of virus attachment. Suramin, a symmetrical polysulfonated naphthylamine that has been used for the treatment of human trypanosomiasis, has been shown to be an antitumor and antiviral agent [17,52,53]. It seems that the inhibitory activity of HS mimetics, including these compounds, is due to their association with GAG binding sites of the putative receptor-binding domain on the DENV E protein [17,24,25,38,39]. These findings are consistent with the interpretation that heparin and HS mimetics are inhibitors of virus adsorption.

A rationale for designing sulfated carbohydrate compounds with low molecular mass as anti-DENV agents targeting E protein functions has been reported [54]. Significant inhibitory activity is exerted by 3-*O*-Sulfated GlcA on DENV2 infection with an EC_50_ value of 120 μM. Two negatively charged groups, 3-*O*-sulfate and 6-C-carboxylic acid, appear to be essential for anti-DENV activity. Docking simulation demonstrated the binding potential of this small compound with respect to DENV E protein, and also showed that the distance and conformation of these negative charges on the carbohydrate may be suitable for association with three responsible amino acid residues of E protein critically involved in virus adsorption. Similar to other HS mimetics, this compound competitively prevents DENV adsorption to host cells.

In most studies on inhibitors of virus-cell surface binding, experiments were carried out at 4°C during coincubation of compounds with cells and virus particles. However, previous studies indicated that DENV particles do not bind efficiently to the cells at this temperature [55,56]. These studies suggest that the virus binding affinity at 4°C is lower than that at physiological temperature, and some inhibitors of virus-cell surface binding might not be properly evaluated. Thus, some inhibitors may block virus infection in the post-virus binding steps, such as virus-endosome membrane fusion. Further careful characterization is required.

### 2.2. Inhibitors of virus-induced membrane fusion

Crystal structure analysis demonstrated that a pocket of the DENV E protein, which is located at a hinge region between domains I and II, is occupied by the ligand, octyl-β-d-glucoside (β-OG) (Figure 2) [24]. Compounds blocking the β-OG pocket are expected to suppress conformational changes of the E protein that are essential for fusion between virus and host endosome membranes (Table 2). Although these compounds are not directly related with carbohydrate molecules, their inhibitory mechanisms are possibly similar to that of β-OG. In understanding the mechanism of the action of β-OG, it is valuable to show the functional properties of these compounds. Therefore, this review also introduces their chemical and biochemical properties.

An *in silico* screen for small molecules could potentially identify candidate molecules capable of binding to the β-OG pocket. Combinatorial computational approaches identified two tetracycline derivatives against flaviviruses [57]. Both compounds were tetracycline derivatives with estimated IC_50 _values of 67.1 μM and 55.6 μM, respectively. Although this study did not utilize fusion assays, the compounds were computationally estimated to interact with critical hydrophobic residues that affect membrane fusion. A combination of high-throughput *in silico* screening with this hydrophobic pocket and evaluation of inhibitory activity by cell-based assays identified compound 6 as a fusion inhibitor with EC_50_ of 119 nM against DENV2 in A549 cells. Mechanism-of-action studies demonstrated that the compound acts in the early step of DENV infection, causing arrest of DENV in vesicles that colocalize with endosomes [58]. Another compound, NITD448 that inhibits DENV fusion reduces viral titers with an EC_50_ of 9.8 μM. Time-of-addition experiments showed that the compound acts via inhibition of fusion [59].

A doxorubicin derivate, SA-17, carrying a squaric acid amide ester moiety at the carbohydrate group was identified as a fusion inhibitor of DENV2 with EC_50_ of 0.52 μM. Docking simulation experiments showed that the compound also associated with amino acid residues critical for membrane fusion, Thr-48, Glu-49, Ala-50, Lys-51, and Gln-52, in the hydrophobic β-OG pocket of the E protein [24,60,61].

**Table 2:** Chemical and antiviral properties of inhibitors of virus fusion

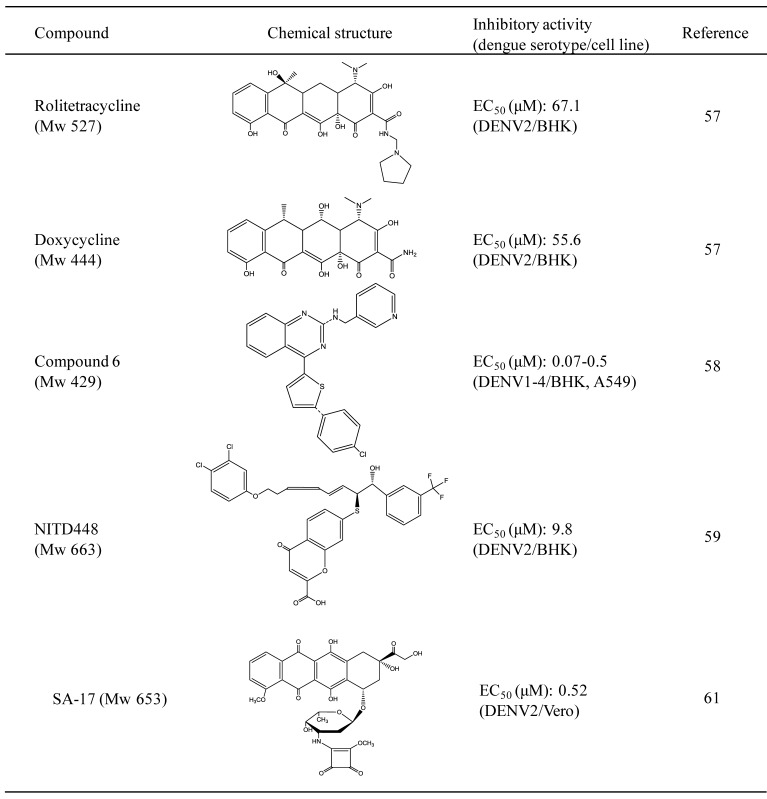

## 3. Conclusion and Future Directions

Repeated infection challenge with different serotypes of DENV increases the risk of antibody-dependent enhancement (ADE) [4,8,9]. Maximal protection to the same extent against all four serotypes with one drug or vaccine is required to control dengue diseases, particularly dengue hemorrhagic disorders. Inhibitors targeting host factors involved in virus entry are interesting targets and may overcome this problem. Accumulating knowledge regarding the processes of DENV entry into the host cell and the recent progress in the *in silico* techniques will contribute to the development of a new class of DENV inhibitors, i.e., entry inhibitors.

While in the *in vitro* assays the affinity of carbohydrate derivatives, including GAG-related compounds, HS mimetics, and compounds non-structurally related to the DENV E protein, will determine their antiviral activity, additional factors, such as physicochemical and pharmacological properties, including membrane permeability and bioavailability, will be critical for the development of effective antiviral drugs for use *in vivo*. Among the inhibitors of virus adsorption, heparin, other GAGs, and sulfated polysaccharides bind to plasma proteins, resulting in significant loss of bioavailability. The smaller size of these compounds relative to other high-molecular weight sulfated polysaccharides tested may account for their greater *in vivo* efficacy based on better bioavailability. In addition to bioavailability, the side effects of HS mimetics due to their anticoagulant activity, restrict their use as antiviral drugs. Thus, in terms of anti-DENV application of virus adsorption inhibitors, it will be critical to identify the minimal determinants for effective antiviral activity of sulfated polysaccharides, including heparin, etc. The smallest molecular weight inhibitor, 3-*O*-sulfated GlcA, may be a useful lead compound that is expected to show low coagulopathy and prolonged bioavailability in addition to effective *in vivo* inhibition of infectivity.

The fusion process is an alternative target for the development of effective *in vivo* antiviral agents. *In silico* virtual screening with *in vitro* assays generated potent anti-DENV agents as described above. Rational screening efforts for fusion inhibitors have continued and generated more potent compounds with better physicochemical and pharmacological properties [62,63,64,65,66,67].

A previous study indicated that peptide entry inhibitors prevent antibody-mediated enhancement of DENV2 infection of human, FcRII bearing K562 cells *in vitro* [68]. In conclusion, entry inhibitors have great potential for use either alone or in combination for treatment of dengue diseases.
